# Simultaneous bilateral mastectomy and RRSO for *BRCA2-*positive non-invasive breast cancer in Japan: a case report and analysis of initial experience

**DOI:** 10.1186/s13053-023-00268-y

**Published:** 2023-11-13

**Authors:** Aya Tanaka, Megumi Matsumoto, Mami Takao, Shoko Miura, Yuri Hasegawa, Ryota Otsubo, Hiroko Hayashi, Ichiro Isomoto, Kiyonori Miura, Takeshi Nagayasu

**Affiliations:** 1 Department of Surgical Oncology, Nagasaki University Graduate School of Biomedical Sciences, 1-7-1 Sakamoto, Nagasaki, 852-8501 Japan; 2 Genetic Counseling Unit, Clinical Genomics Center, Nagasaki University Hospital, Nagasaki, Japan; 3 Department of Obstetrics and Gynecology, Nagasaki University Graduate School of Biomedical Sciences, Nagasaki, Japan; 4 Department of Pathology, Nagasaki University Graduate School of Biomedical Sciences, Nagasaki, Japan; 5 Department of Pathology, Sasebo City General Hospital, Nagasaki, Japan; 6 Department of Radiology, St. Francis Hospital, Nagasaki, Japan

**Keywords:** Hereditary breast and Ovarian cancer, *BRCA1*, *BRCA2*, Contralateral risk-reducing mastectomy, Risk-reducing salpingo-oophorectomy

## Abstract

**Background:**

In Japan, genetic testing, surveillance, and risk-reducing surgery for hereditary breast and ovarian cancer (HBOC) syndrome have been covered by the Japanese national insurance system since April 2020. On the other hand, the current situation is that medical care, including surveillance of undiagnosed (cancer-free) patients, is self-funded even for individuals with HBOC. We report a case in which breast cancer was diagnosed at an early stage during surveillance for cancer-free HBOC at the patient’s own expense, and risk-reducing surgery was performed at the same time as treatment for breast cancer.

**Case presentation:**

The patient was a 63-year-old woman. Her sister had a history of breast cancer in her 30s and was found to be a *BRCA2* pathogenic variant carrier by genetic testing. The patient therefore presented to the genetic department of our hospital and underwent genetic testing (out-of-pocket). A pathogenic variant was found at the same site. During annual breast and ovarian surveillance at the patient’s own expense, a physician with sufficient expertise in contrast-enhanced breast magnetic resonance imaging (MRI) noticed a change in the contrast enhancement pattern on breast MRI and performed needle biopsy, revealing ductal carcinoma in situ. At the request of the patient, she underwent concurrent contralateral risk-reducing mastectomy and risk-reducing salpingo-oophorectomy in addition to breast cancer treatment.

**Conclusions:**

We encountered a case in which cancer treatment and risk-reducing surgery were performed at the same time for a pathogenic variant carrier who was very anxious about developing cancer. Surveillance of cancer-free *BRCA1/2* mutation carriers and expansion of insurance coverage for surgery are important future issues.

## Background

Breast cancer is the most common cancer among Japanese women. Genetic factors are associated with the development of breast cancer in 5–10% of all breast cancer patients [1]. Hereditary breast and ovarian cancer (HBOC) is a syndrome of predisposition to cancer development, including breast cancer and ovarian cancers, caused by germline pathogenic variants of *BRCA1* or *BRCA2* (g*BRCA1/2*-positive). Several studies have found that the cumulative risk of developing breast cancer by 70 years of age is 55–72% in women with *BRCA1* mutations and 45–69% in women with *BRCA2* mutations, whereas the cumulative risk of breast cancer in the general population is 12%. The cumulative risk of developing ovarian cancer has been reported as 39–44% for *BRCA1* mutations and 11–17% for *BRCA2* mutations, compared to a cumulative risk of ovarian cancer in the general population of 1–2% [2]. In Japan, 1.45% of breast cancer cases reportedly involve a *BRCA1* pathogenic variant and 2.71% have some *BRCA2* pathogenic variant [3]. Until a few years ago, all medical procedures, such as *BRCA1/2* genetic testing and some risk-reducing surgeries, were paid for out-of-pocket by patients. A review of medical fees in Japan was implemented in April 2020. The BRACAnalysis test (Myriad Genetics, Salt Lake City, UT, USA) was covered for breast cancer patients who met certain conditions and for all ovarian cancer patients [4, 5]. Both risk-reducing salpingo-oophorectomy (RRSO) and risk-reducing mastectomy (RRM) are covered for g*BRCA1/2*-positive breast and ovarian cancer patients [4, 6]. Surveillance breast magnetic resonance imaging (MRI) may also be covered for *BRCA1/2* pathogenic variant carriers not undergoing risk-reducing surgery [4]. We conduct breast surveillance for *BRCA1/2* pathogenic variant carriers in collaboration with hospitals that have specialists with sufficient expertise in contrast-enhanced MRI of the breast and centers that can perform MRI-guided biopsies.

However, these benefits are only available to those who have already developed breast or ovarian cancer, and the current situation is that surveillance and preventive care for HBOC remains an out-of-pocket expense for those who have not developed cancer [7]. We report on a patient undergoing out-of-pocket surveillance as an undiagnosed (cancer-free) HBOC who was subsequently diagnosed with non-invasive breast cancer and was able to receive breast cancer treatment and risk-reducing surgery simultaneously under insurance coverage.

## Case presentation

**Fig. 1:**
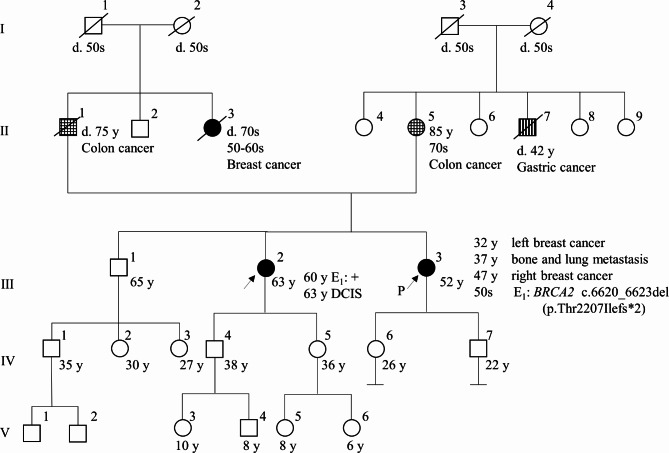
Family history of the pedigree. II-1: Died of colon cancer at 75 years old. II-3: Died of breast cancer in her 70s. II-5: Diagnosed with colon cancer in her 70s. II-7: Died of gastric cancer at 42 years of age. III-2: Diagnosed with ductal carcinoma in situ (DCIS) after single-site analysis of the same *BRCA2* pathogenic variant (E1) as her younger sister (III-3). III-3: Found to be a carrier of the *BRCA2* pathogenic variant (E1) by BRACAnalysis in her 50s

**Fig. 2:**
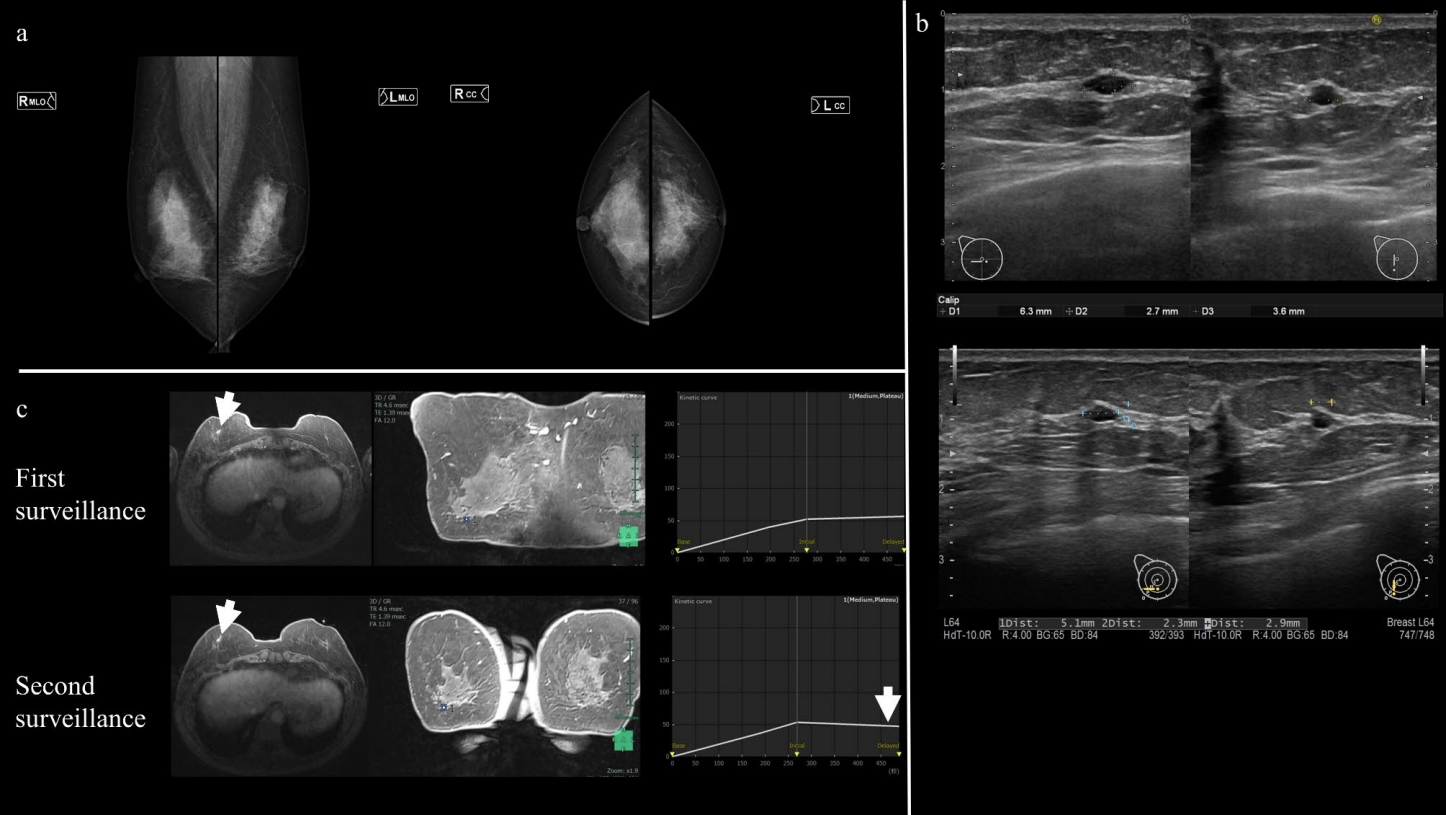
Breast imaging. (**a**) Mammography shows no malignant findings. Bilateral diffuse microcircular calcifications are observed in both breasts, but no significant differences from the previous imaging are apparent. The case is classified as category 2. (**b**) Breast echography shows a well-defined nodule in the lower outer quadrant on right breast surveillance before starting breast surveillance (top; approximately 2 years and 10 months previously) and before performing core needle biopsy (bottom). After 2 years and 10 months, almost no change is evident. (**c**) Contrast-enhanced MRI of the breast taken during the first surveillance (top) and the second surveillance (bottom). Both MRIs show similar nodules in the right breast, but washout is present during the second surveillance that is not seen on the kinetic curve from the first surveillance (white arrow)

**Fig. 3:**
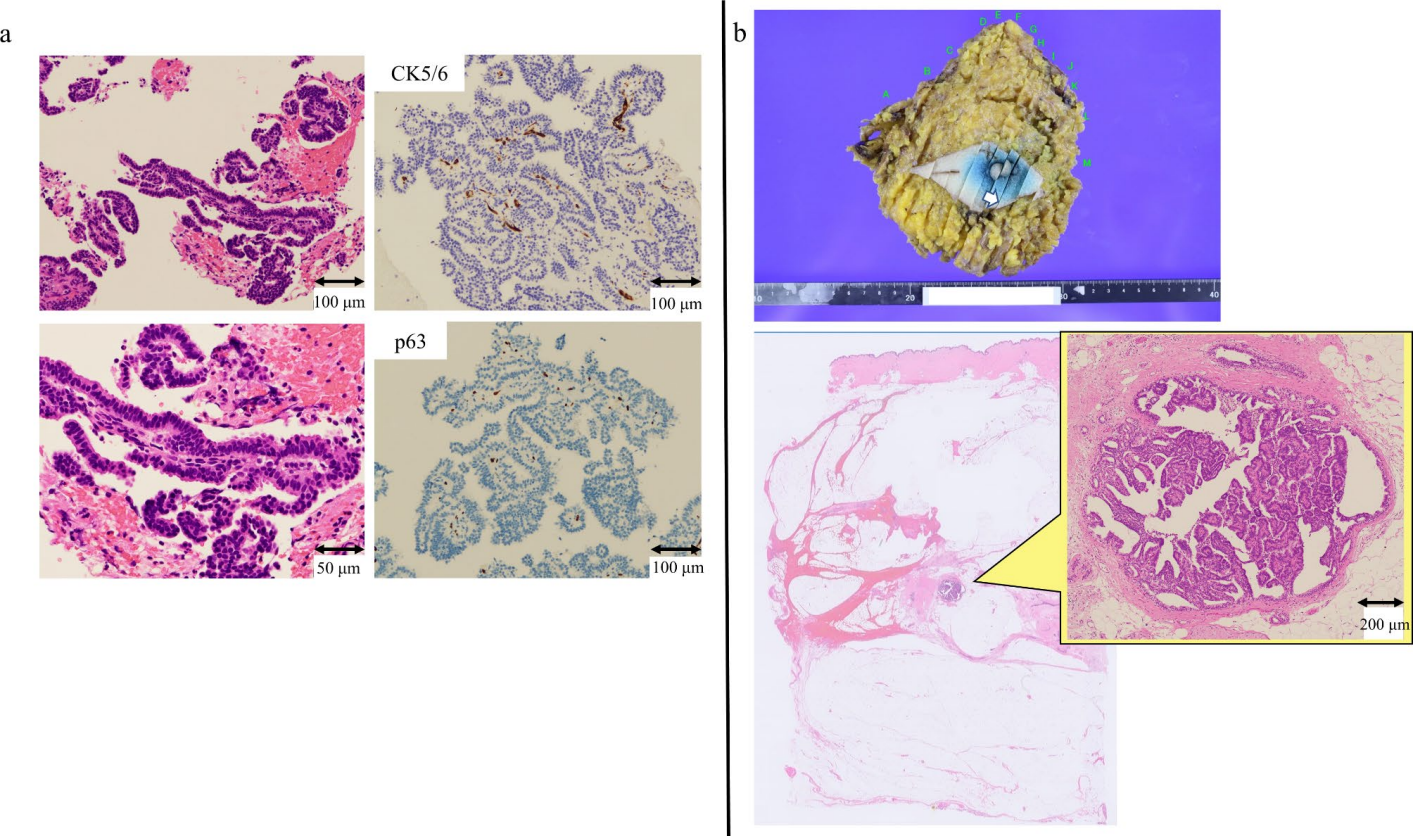
Pathologic findings. (**a**) Results of 14-G core needle biopsy. Tumor cells with densely stained nuclear chromatin show papillary growth in the mammary ducts. The glandular epithelium has few lining myoepithelial cells. A few myoepithelial cells show positivity for p63. CK5/6 is negative in proliferating glandular epithelium (positive cells are myoepithelial cells). The diagnosis is DCIS. (**b**) Macroscopic appearance (top) and microscopic appearance (bottom). Loupe image (bottom left) and high magnification image of the lesion within it (bottom right). Postoperative histopathological diagnosis reveals that atypical cells have proliferated within the duct to a diameter of 1.7 mm, leading to the diagnosis of low-grade DCIS. Arrow in macroscopic appearance: site at which non-invasive cancer is confirmed

A 64-year-old woman (III-2) presented to our clinic for single-site analysis of a *BRCA2* pathogenic variant that had been identified in her sister (III-3). Her family tree (Fig. 1) showed that her paternal aunt (II-3) had been diagnosed with breast cancer in her 50s, and her younger sister (III-3) had developed breast cancer in her 30s. Since discovering the presence of the pathogenic variant, the patient has undergone regular out-of-pocket ovarian and breast surveillance every 6–12 months. The price of out-of-pocket surveillance ranges from about $340 USD to $480 USD (including tax, estimated at the September 2023 exchange rate) per visit, depending on the imaging tests performed. About a year and a half into this surveillance, a nodule displaying a different contrast pattern from previous results was noted on contrast-enhanced MRI of the breast and we decided to perform a detailed evaluation at our hospital. Mammography showed no malignant findings (Fig. 2a), and breast echography showed a well-defined nodule measuring 6 mm in diameter in the lower outer quadrant of the right breast (Fig. 2b). Contrast-enhanced MRI of the breast also showed a nodule similar to the previous one in the right breast, but washout that had not been seen on the previous kinetic curve was identified (Fig. 2c). Because very early breast cancer, such as ductal carcinoma in situ (DCIS), could not be ruled out, the patient was referred to our department. Since she was at high risk for developing breast cancer as a carrier of a *BRCA2* pathogenic variant, we performed core needle biopsy of the small breast mass with a 14-G needle after injecting local anesthetic subcutaneously and around the tumor under ultrasonographic guidance. The pathology report of the core needle biopsy identified DCIS, a so-called non-invasive ductal carcinoma of the breast (Fig. 3a). In addition to right breast cancer, hereditary breast cancer and ovarian cancer syndrome was identified. We therefore conferred with the patient and decided to perform breast cancer surgery, contralateral risk-reducing mastectomy (CRRM), and RRSO. First, carbon dioxide pneumoperitoneum was started and gynecologists in our hospital performed laparoscopic RRSO in the lithotomy position after the legs were separated, elevated, and supported by leg holders. Afterwards, carbon dioxide pneumoperitoneum was terminated, then the breast surgeon performed breast surgery with the patient in the supine position (right mastectomy and sentinel lymph node biopsy, CRRM for the left breast). Breast reconstruction was not performed. Total operation time was 6 h and 2 min, including the time for setting the surgical environment. This patient showed good food intake from the day after surgery and was discharged home on postoperative day 6. Postoperative histopathological diagnosis revealed that atypical cells had proliferated within the duct to 1.7 mm in diameter, leading to the diagnosis of low-grade DCIS (Fig. 3b). In this case, we were able to resect the cancer at pStage 0. No malignant findings were observed in the contralateral breast or bilateral fallopian tubes and ovaries.

Currently, the patient visits our hospital about once every 6 months for peritoneal cancer surveillance and postoperative wound examination. In the future, we plan to have the Department of Obstetrics and Gynecology perform peritoneal cancer surveillance, such as computed tomography, and to have the patient perform regular self-examination of the chest (focusing on the surgical scars of the breast).

## Discussion and conclusions

In g*BRCA1/2*-positive variant carriers, RRSO has been confirmed to reduce the risk of developing cancer and prolongs overall survival [5, 8–13]. Risk-reducing surgery not only contributes to lower mortality in HBOC patients, but also has a clinically significant impact in reducing the fear of future cancer [14]. In Japan, with the April 2020 Physician Fee Review, certain breast cancer patients and all ovarian cancer patients have become able to receive the BRACAnalysis test (Myriad Genetics) as a part of covered medical treatment [4, 5]. In addition, RRSO and RRM are covered for g*BRCA1/2*-positive breast and ovarian cancer patients [4, 6], and surveillance MRI of the breast is also covered for g*BRCA1/2*-positive breast and ovarian cancer patients not undergoing risk-reducing surgery [4]. As of the end of 2022, about 200 cases of genetic counseling for HBOC have been performed in our hospital. *BRCA1/2* genetic testing was performed in approximately 160 cases, with the pathogenic variant found in 20 cases. More than 80% of tests have been performed in the period since insurance coverage was approved. We have also performed 8 RRSOs in our hospital. In all cases of RRSO performed, breast cancer was already present (Table 1). The number of RRSO and RRM procedures performed has increased since coverage by health insurance was obtained.

**Table 1:** Cases of risk-reducing surgery performed at our hospital through March 2022

Case	Variant	Age at first onset of breast cancer (years)	Age at diagnosis of HBOC (years)	Age at RRSO (years)	Age at CRRM (years)	Payment for risk-reducing surgery
1	*BRCA1*	28	41	42	not performed	self-funded
2	*BRCA1*	39	39	40	40	insurance
3	*BRCA1*	41	43	43	not performed	insurance
4	*BRCA1*	48	48	49	53	self-funded
5	*BRCA1*	50	50	51	not performed	insurance
6	*BRCA2*	43	46	47	not performed	insurance
7	*BRCA2*	57	59	60	not performed	insurance
8	*BRCA2*	64	63	64	64	insurance

*Note*: Most cases underwent surgery after obtaining insurance coverage, but in some cases risk-reducing surgery was performed on a self-funded basis. All cases of RRSO have been performed so far, all within one year of HBOC diagnosis. CRRM was also performed in 3 cases, as the procedure was covered by insurance. Case 2 underwent breast cancer surgery and CRRM at the same time, followed by reconstruction, then RRSO after 2 months. In Case 3, RRSO revealed cancer in the ovary and fallopian tube, and radical surgery was performed later. Stage II ovarian cancer was diagnosed. This time, we introduce the eighth case, representing the first case in which breast surgery and ovarian/fallopian tube surgery were performed on the same day in our hospital

Specifically for RRSO, we work with the Department of Obstetrics and Gynecology to ensure that RRSO is performed within one year of HBOC diagnosis. If CRRM and RRSO can be performed at the same time as breast cancer treatment, many patient anxieties can be expected to be alleviated. However, close cooperation with the Department of Obstetrics and Gynecology and Department of Anesthesiology is necessary, and addressing multiple issues simultaneously is challenging. In the present case, after discussion with our Department of Obstetrics and Gynecology, we were able to perform all surgeries on the same day. On reflection, the surgery took somewhat longer than usual for this type of surgery. Currently, bilateral mastectomies are performed by one surgeon at our hospital, but if two surgeons could perform bilateral mastectomies at the same time, operation time could be reduced. We will continue to consider other ways to shorten operation time, both in terms of technique and operating room environment. The average hospital stay after breast surgery in Japan tends to be longer than in Western countries. As such, we will also consider ways to promote earlier discharge from hospital.

Generally, advanced ovarian cancer has a poor prognosis. In particular, HBOC-related ovarian cancer is often advanced at the time of diagnosis and shows poor prognosis [15, 16]. Previous reports have shown that ovarian cancers associated with g*BRCA* pathogenic variants exhibit distinct clinical behaviors, including advanced stage (International Federation of Gynecology and Obstetrics (FIGO) stages III–IV [17]), papillary, high-grade serous, and poorly differentiated adenocarcinoma. As a result, performing risk-reducing surgery for HBOC cases in which ovarian cancer has not yet developed can significantly impact life prognosis. Risk-reducing surgery can thus be considered cancer-preventive medicine in the true sense of the term. However, in Japan, risk-reducing surgery is not covered by health insurance for patients without cancer [7]. At our hospital, CRRM costs about $4,200 USD (including tax), RRSO costs about $6,000 USD (including tax), and hysterectomy and ovarian salpingectomy costs about $8,300 USD (including tax). More than ever, we are calling for the extension of HBOC insurance coverage for people who have not yet developed cancer.

The patient in this case had been undergoing surveillance at her own expense since she had been found to be g*BRCA2*-positive, due to concerns about developing ovarian cancer. While the National Comprehensive Cancer Network (NCCN) guidelines recommend risk-reducing surgery for g*BRCA1/2*-positive variant carriers, they do not recommend ovarian surveillance. This is because no evidence suggests that such surveillance has any impact in reducing the risk of developing, or dying from, cancer. However, because the patient had not developed cancer, she would have had to pay out-of-pocket for any tests or risk-reducing surgeries. She was therefore reluctant to undergo risk-reducing surgery because of the high cost, and chose instead to undergo breast and ovarian surveillance rather than risk-reducing surgery. Surveillance and testing led to a diagnosis of non-invasive breast cancer, at which time both breast cancer surgery and risk-reducing surgery were covered by the Japanese health insurance system. Because few changes were evident on imaging and only slight changes in the kinetic curve were seen on contrast-enhanced MRI of the breast, we might not have considered core needle biopsy in the absence of a background of pathogenic mutations in g*BRCA2*. After the surgery, the patient was very satisfied because she had been able to undergo surgery for the cancer and RRSO and CRRM at the same time. Because this was before any ovarian cancer developed, she was able to undergo laparoscopic surgery and felt very well after the surgery. The patient has an adult daughter who does not want to be tested for *BRCA1/2*. In the past, she had left it up to her daughter to decide whether to undergo *BRCA1/2* testing, but now that she has undergone this surgery, she is actively encouraging her daughter to undergo testing. If tested, her daughter would know her own susceptibility and could better plan for her healthcare. If she discovers she has a *BRCA2* pathogenic variant that had been identified in her mother, close surveillance might detect early breast and ovarian cancer. Even with these benefits, however, cancer-free individuals may be reluctant to be tested for *BRCA1/2* if surveillance and risk-reducing surgery must be self-funded. Unfortunately, this is the current state of HBOC medicine in Japan.

In conclusion, we encountered a case of HBOC in which both RRSO and RRM were performed simultaneously in addition to breast cancer treatment at the request of the patient. When HBOC was diagnosed, the patient was concerned about developing ovarian cancer, but since she had not developed cancer, the cost of risk-reducing surgery was very high. This was therefore a case in which risk-reducing surgery could not be performed. We believe that extending insurance coverage for HBOC cases to those patients who have not yet developed cancer represents an extremely important issue for true cancer-preventive medicine in the future. In addition, if risk-reducing surgery is not covered by insurance because no cancer has yet developed, the key issue is how to detect cancer early and prevent a life-threatening status through regular surveillance performed at personal expense. To improve the accuracy of breast and ovarian surveillance, collaboration needs to be strengthened between breast oncologists and specialists with sufficient expertise in contrast-enhanced MRI of the breast and gynecologic oncologists, to create a safe environment not only for cancer patients, but also for cancer-free HBOC individuals.

## Data Availability

Raw data were generated at Nagasaki University Graduate School of Biomedical Sciences. Derived data supporting the findings of this study are available from the corresponding author (AT) upon reasonable request.

## Abbreviations

HBOC: Hereditary breast and ovarian cancer
CRRM: Contralateral risk-reducing mastectomy
RRSO: Risk-reducing salpingo-oophorectomy
MRI: Magnetic resonance imaging
DCIS: Ductal carcinoma in situ
FIGO: International Federation of Gynecology and Obstetrics
NCCN: National Comprehensive Cancer Network
